# Medical‐Legal Partnerships as Structural Interventions: Integrating Implementation Science and Community‐Engaged Research to Advance HIV Care

**DOI:** 10.1002/jia2.70191

**Published:** 2026-10-08

**Authors:** Omar Martinez, Edda Santiago Rodriguez, Guillermo Prado, Kathryn M. Nowotny, Robin Davison, Cui Yang, Samantha Morton, Ashley French, Souhail Malave‐Rivera, Carlos E. Rodriguez‐Diaz, Florence Momplaisir, Elena Cyrus, Mallory O. Johnson, Miguel Munoz‐Laboy

**Affiliations:** ^1^ University of Central Florida Orlando Florida USA; ^2^ University of Puerto Rico San Juan Puerto Rico USA; ^3^ University of Miami Coral Gables Florida USA; ^4^ Rutgers University School of Public Health Piscataway New Jersey USA; ^5^ Samantha Morton Consulting Boston Massachusetts USA; ^6^ Boston University School of Public Health Boston Massachusetts USA; ^7^ University of Pennsylvania Philadelphia Pennsylvania USA; ^8^ University of California San Francisco San Francisco California USA; ^9^ Stony Brook University Stony Brook New York USA

**Keywords:** CFIR, community engagement, health equity, health‐harming legal needs, HIV care continuum, implementation outcomes, interdisciplinary collaboration, legal services integration, medical‐legal partnerships, RE‐AIM

## Abstract

**Introduction:**

People living with HIV (PLWH) often face health‐harming legal needs (HHLNs), including housing instability, discrimination, immigration concerns, benefit denials and interpersonal violence, which can undermine engagement in care and health outcomes. Medical‐legal partnerships (MLPs) integrate legal services into healthcare settings to address these drivers of health inequities and improve access to HIV prevention and care.

**Methods:**

This study presents mixed‐methods implementation findings from three federally funded MLP initiatives serving PLWH in New York City, Philadelphia, Orlando and San Juan. Implementation was evaluated using the Reach, Effectiveness, Adoption, Implementation, and Maintenance (RE‐AIM) framework and the Consolidated Framework for Implementation Research (CFIR), with guidance from Community and Scientific Advisory Boards. Quantitative data were derived from standardized HHLN screening tools, while qualitative interviews with clients, healthcare providers and legal professionals explored implementation experiences, contextual factors and perceived impacts.

**Results:**

Across the three initiatives, 928 participants were enrolled between 2022 and 2026. Core intervention components included: (1) workforce training on HHLNs and legal‐health integration; (2) routine legal screening; (3) referral to community‐based legal services; and (4) co‐located legal clinics and preventive legal education. Common HHLNs included housing instability, benefit denials, immigration concerns, criminal record expungement, advance care planning and discrimination. Implementation demonstrated strong feasibility, acceptability, adoption and cultural responsiveness across sites. Participants described reductions in anxiety, improved confidence, greater trust in healthcare systems and improved engagement in HIV care following legal support. Trainings co‐developed with community stakeholders enhanced providers’ understanding of structural determinants and legal remedies, while interdisciplinary collaboration reduced provider burden and strengthened care coordination. RE‐AIM findings demonstrated broad reach and organizational adoption, and CFIR analyses identified leadership engagement, organizational readiness and client‐provider trust as key implementation facilitators. Cross‐study findings informed development of the equity‐centred implementation framework for MLP in HIV care, integrating implementation science, health equity and community engagement principles to guide future adaptation and scale‐up.

**Conclusions:**

MLPs are a feasible, equity‐focused and scalable strategy for addressing structural barriers that affect HIV outcomes. Findings support continued investment in legal‐health integration, workforce development and community‐engaged approaches to strengthen HIV care delivery and advance health equity among populations disproportionately affected by HIV.

## 1 Introduction

People living with HIV (PLWH) frequently face intersecting structural barriers that impede their health, wellness and sustained engagement across the HIV care continuum [1, 2]. These barriers often manifest as health‐harming legal needs (HHLNs), defined as unmet legal problems that negatively affect a person's health or access to healthcare [3, 4, 5, 6]. HHLNs encompass a range of social and structural determinants of health, such as housing instability and unsafe living conditions, denial or termination of public benefits, employment or healthcare discrimination, immigration‐related challenges and exposure to interpersonal or gender‐based violence [7, 8, 9]. Traditional biomedical and behavioural interventions, while critical to HIV prevention and care, often overlook these upstream determinants that directly shape individual and community health outcomes. Achieving and sustaining viral suppression requires approaches that extend beyond the clinical setting and integrate legal, social and structural expertise into care delivery.

Mounting evidence indicates that HHLNs, including disputes over insurance coverage, housing instability and related legal vulnerabilities (e.g. eviction risk, landlord disputes, unsafe housing conditions and housing discrimination), unsafe working environments and unresolved civil or immigration matters, are well‐documented barriers to accessing, engaging in and maintaining HIV care [8, 9, 10, 11, 12]. These legal and structural barriers disproportionately affect low‐income and underserved PLWH, particularly racial and ethnic minority communities, and further strain already overburdened health systems. Addressing HHLNs through integrated, cross‐sector, community‐centred and structural interventions is essential to advancing equitable outcomes across the HIV care continuum and achieving the broader goals of Ending the HIV Epidemic [13, 14, 15, 16].

Medical‐legal partnerships (MLPs) are a promising structural intervention that embeds civil legal services into healthcare and social service settings [9, 17, 18, 19, 20, 21, 22, 23, 24, 25]. Since their emergence in 1993, MLPs have demonstrated promise in improving access to benefits, stabilizing housing and addressing legal barriers that undermine health [24, 26, 27, 28, 29, 30, 31, 32, 33, 34, 35, 36, 37, 38]. By equipping providers to identify legal needs and connecting patients to trusted legal partners, MLPs can help resolve pressing crises while mitigating long‐term structural inequities [21, 39]. MLPs are grounded in health equity principles and emphasize culturally responsive, client‐centred and community‐driven approaches [21, 40, 41, 42]. As such, they have potential to improve clinical outcomes and reduce disparities in HIV incidence, care engagement and viral suppression.

Implementation science frameworks, such as Reach, Effectiveness, Adoption, Implementation, and Maintenance (RE‐AIM) and Consolidated Framework for Implementation Research (CFIR) [43], offer a promising approach for evaluating the impact of structural interventions such as MLPs [44]. Rather than focusing solely on individual behaviour change, these frameworks emphasize the broader organizational, contextual and system‐level factors that shape intervention uptake and long‐term impact. In the context of MLPs, implementation science offers a way to examine how legal advocacy, cross‐sector collaboration and institutional partnerships may influence health outcomes, healthcare engagement and health equity. This perspective is particularly important for advancing structural approaches that address the root causes of health disparities and for generating evidence needed to support broader adoption and sustainability of MLP models across healthcare systems.

This study presents findings from three federally funded initiatives designed to establish and evaluate MLPs for PLWH in New York City, NY, Philadelphia, PA, Orlando, FL and San Juan, PR. We integrate implementation science frameworks, including the RE‐AIM and CFIR frameworks, with community‐engaged research approaches to assess the feasibility, acceptability and effectiveness of these interventions.

## 2 Methods

### 2.1 Study Design and Setting

This study examines three federally funded MLP initiatives implemented across four U.S. jurisdictions: New York City, NY, Philadelphia, PA, Orlando, FL and San Juan, PR. See Table 1. Each was led by academic institutions in collaboration with primary care centres, HIV service organizations and long‐standing legal aid partners. These legal partners, including organizations such as the Legal Clinic for the Disabled (Philadelphia), LegalHealth (New York City), Servicios Legales de Puerto Rico (San Juan) and Community Legal Services (Orlando), have deep histories of serving medically underserved and marginalized communities. Legal services were made accessible through affiliated legal generalists, attorneys equipped to address a broad range of common civil legal needs and provide initial legal assessment, guidance, referrals and coordination, embedded within clinics and supported by paralegals and robust pro bono attorney networks. This structure enabled “warm handoffs” between clinicians and attorneys and facilitated timely legal consultations during routine care. Sites piloted MLP interventions tailored to local needs while maintaining core components for cross‐site comparison. Guided by a community‐engaged framework, the research team identified effective implementation strategies, built interdisciplinary partnerships, leveraged community resources and developed mechanisms to support the scale‐up of critical‐time legal interventions, a time‐limited, intensive legal care approach designed to stabilize individuals during periods of transition and heightened vulnerability. All study sites are large metropolitan areas with high HIV prevalence among medically underserved populations, disproportionately affecting formerly incarcerated individuals, Latinx groups and immigrants facing intersecting epidemics of infectious and chronic disease.

**TABLE 1:** MLP studies.

Study	Population and setting	Study design and sample	Aim 1	Aim 2	Aim 3/exploratory aim	Frameworks and key components
**MLP 1: Formerly incarcerated individuals living with HIV** *(Orlando, Florida)*	This study is currently being conducted at a large community‐based health centre in Orlando, Florida, serving formerly incarcerated individuals with HIV, a population among the most medically underserved and stigmatized.	A pilot before‐and‐after trial evaluated patient health, functioning and linkage to HIV care, retention, and viral suppression among formerly incarcerated individuals living with HIV. Sample: *n* = 100	Finalize protocols and develop a comprehensive medical‐legal partnership (MLP) training manual for health, legal and social service providers. A Community Advisory Board, Scientific Advisory Board and Legal Expert Panel informed development of these materials.	Assess the feasibility and acceptability of a critical time intervention‐medical‐legal partnership (CTI‐MLP) model to improve HIV continuum outcomes among formerly incarcerated individuals living with HIV. Outcomes included patient health, functioning, linkage to HIV care, retention in care and viral suppression.	Identify the most efficient mechanisms for CTI‐MLP delivery using indicators from the RE‐AIM and CFIR frameworks to assess facilitators and barriers to implementation.	Frameworks: RE‐AIM; CFIR Key components: critical time intervention model; integrated legal services; provider training; community‐engaged implementation; HIV care linkage and retention support
**MLP 2: Immigrant and Latinx communities** *(San Juan, Puerto Rico; New York City, NY; Philadelphia, PA)*	Building on prior NIH‐funded MLP studies in HIV care (1R21MH115820; R34MH125718), this study developed and is currently evaluating the critical time intervention‐medical‐legal partnership (CTI‐MLP) model designed to address social and legal determinants of health within primary care settings. The study targeted Latinx and migrant communities disproportionately affected by HIV, chronic diseases and structural inequities, yet among the most medically underserved populations in the United States and its territories.	A hybrid type II effectiveness‐implementation trial used a cluster‐randomized design across six predominantly Latinx‐serving primary care centres in New York City, Philadelphia and San Juan. Sample: *n* = 960	Evaluate patient‐level outcomes by testing the effectiveness and cost‐benefit of CTI‐MLP in improving patient‐reported global health and clinical engagement in primary care. Primary hypotheses were that primary care centres implementing CTI‐MLP would demonstrate higher global health ratings and greater retention in treatment at 3‐, 6‐, 9‐ and 12‐month follow‐ups compared to standard care.	Examine implementation outcomes by identifying efficient mechanisms for CTI‐MLP delivery, guided by RE‐AIM and CFIR frameworks to evaluate factors influencing reach, adoption, implementation and sustainability. The study also evaluated whether CTI‐MLP demonstrated higher cost‐effectiveness and a favourable cost‐benefit ratio at 12 months.	Develop innovative community engagement strategies to ensure sustainability of MLP integration. Using a community‐engaged framework for change, the study sought to identify dissemination, implementation and sustainability mechanisms to support CTI‐MLP scale‐up across community health centres.	Frameworks: RE‐AIM; CFIR; community‐engaged dissemination and sustainability approaches Key components: cluster‐randomized implementation; integrated legal and healthcare services; cost‐benefit and cost‐effectiveness analyses; sustainability and scale‐up strategies; community‐engaged implementation
**MLP 3: People living with HIV** **Organizational Partnerships for Healthy Living (OPaHL)** *(Philadelphia, Pennsylvania)*	The Organizational Partnerships for Healthy Living (OPAHL) initiative was a multilevel, community‐informed intervention implemented within two HIV service organizations in Philadelphia. The intervention integrated legal expertise into healthcare delivery to improve outcomes for PLWH.	A cluster‐randomized pilot trial was conducted across two HIV treatment organizations. One site was randomized to the OPAHL intervention, which included integrated legal support, while the comparison site received standard care with external legal aid referral. Intervention site: *n* = 95 PLWH Comparison site: *n* = 107 PLWH	Refine the OPAHL intervention prototype for PLWH with detectable viral loads using intervention mapping, a six‐step ecological and multilevel implementation approach. A Community–Scientific Collaborative Board guided adaptation of the OPaHL package, protocols and assessments for implementation.	Test the feasibility, acceptability and preliminary effect sizes of OPAHL through a cluster‐randomized pilot trial comparing integrated legal support to standard care with external legal aid referral.	Exploratory aim: Examine how MLP models can adapt to intersecting crises by exploring opportunities for MLP approaches to address health and legal challenges arising from the intersection of HIV and COVID‐19.	Frameworks: intervention mapping; ecological and multilevel implementation approaches Key components: integrated legal expertise within HIV care; staff training on HIV care and legal needs; case management training on the legal care continuum; co‐location of legal services; communication protocols emphasizing patient autonomy

Abbreviations: CFIR, Consolidated Framework for Implementation Research; CTI, critical time intervention; CTI‐MLP, critical time intervention‐medical‐legal partnership; HIV, human immunodeficiency virus; MLP, medical‐legal partnership; NIH, National Institutes of Health; OPaHL, Organizational Partnerships for Healthy Living; PLWH, people living with HIV; RE‐AIM, Reach, Effectiveness, Adoption, Implementation, and Maintenance.

### 2.2 Intervention Components

Across sites, MLP interventions shared core components while allowing local adaptation. Multidisciplinary training equipped healthcare and legal staff to identify and address HHLNs, emphasizing legal determinants of health, HIV‐related legal issues, screening and referral processes, implementation skills and cultural humility. Standardized HHLN screening was integrated into routine care, facilitating timely identification of housing instability, benefit denials, immigration concerns and discrimination. Trusted community‐based legal partners provided co‐located legal consultations, advocacy and representation, enabling warm handoffs and coordinated care. Preventive legal education workshops further promoted legal literacy and proactive problem‐solving. Together, these components created an equity‐focused model for integrating legal services into healthcare and addressing structural barriers that affect HIV outcomes.

### 2.3 Rationale for Selection of Implementation Strategies

Implementation strategies were selected through an iterative, community‐engaged process informed by implementation science, prior NIH‐funded pilot work and collaboration with healthcare providers, legal partners, community advisory boards, and PLWH. Guided by RE‐AIM and CFIR, strategies were designed to address unmet HHLNs, strengthen healthcare‐legal integration and improve HIV care engagement while maximizing reach, adoption, fidelity and sustainability. Core strategies, including provider training, standardized legal screening, warm handoffs, co‐located legal services, interdisciplinary coordination, preventive legal education and community engagement, were maintained across sites, with local adaptations to reflect differences in patient populations, organizational capacity and policy context.

### 2.4 Data Collection and Analysis

Data were collected from multiple sources to evaluate implementation processes and outcomes across sites. Quantitative measures included standardized HHLN screening tools tailored to each population, capturing issues such as criminal record expungement, voting rights restoration, housing stability, re‐entry support, discrimination, public benefit denials, advance care planning and immigration‐related legal needs (e.g. naturalization, asylum, U‐visas and adjustment of status). The standardized legal problem macro categories used to classify HHLNs across study sites are presented in Supplement A. Additional measures assessed mental health, HIV care continuum outcomes, physical health and quality of life. Staff surveys, informed by the RE‐AIM and CFIR frameworks [45], assessed readiness, adoption and sustainability. All quantitative data were collected using REDCap and analysed descriptively to characterize HHLNs and implementation outcomes. Qualitative data were collected through interviews and open‐ended assessments with clients, healthcare providers and legal professionals. Questions explored perceptions of feasibility, acceptability, implementation experiences and perceived impacts of MLPs. Data were analysed using reflexive thematic analysis [46], with coding guided by RE‐AIM and CFIR constructs as well as emergent themes. Three analysts independently coded transcripts, resolving discrepancies through consensus discussions with senior investigators. Themes were organized to identify cross‐cutting facilitators, barriers and lessons learned related to MLP implementation and impact. Attorney case summaries were also analysed using qualitative coding methods to identify recurring legal issues and service patterns. Detailed analytic procedures have been described elsewhere [8]. The standardized Attorney Case Summary form used by legal partners to document legal needs, legal services delivered, referrals, case status and perceived client outcomes is provided in Supplement B.

### 2.5 Development of the Equity‐Centred Implementation Framework for Medical‐Legal Partnerships in HIV Care

The equity‐centred implementation framework for MLP in HIV care was developed to guide the evaluation of MLP implementation, effectiveness, equity and sustainability across HIV care settings. Consistent with Nilsen's typology [47], the framework functions as both a determinant and evaluation framework by integrating contextual factors, implementation processes and outcomes. Framework development was informed by mixed‐methods data from three federally funded MLP studies, including HHLN screenings, implementation and clinical outcomes, attorney case summaries, qualitative interviews and cross‐site implementation experiences. Development followed an iterative process. First, the investigative team synthesized findings across studies and reviewed relevant implementation science, health equity and structural intervention frameworks. Second, leadership teams from all three MLPs participated in consultation and consensus‐building activities. Third, the framework was refined through interdisciplinary analytic meetings and two multi‐site implementation symposia in Puerto Rico involving investigators, legal aid organizations, healthcare providers and community partners from Orlando, San Juan, New York City and Philadelphia. An agenda from one of the Tri‐City Symposia that supported framework refinement through structured stakeholder discussions and consensus‐building activities is provided in Supplement C. These activities identified shared implementation processes, contextual determinants, barriers, facilitators, adaptation strategies and lessons learned, resulting in consensus on core framework domains, equity‐centred components and evaluation priorities. Data collection for MLP 1 and MLP 2 remains ongoing, and preliminary findings are presented herein, while MLP 3 data collection was completed in 2025.

### 2.6 Implementation Science Frameworks

Evaluation across the three MLP studies was guided by the RE‐AIM and CFIR implementation science frameworks [45, 48]. Together, these frameworks provided a comprehensive approach for assessing implementation outcomes and the contextual factors influencing intervention success and sustainability. RE‐AIM was used to evaluate reach, effectiveness, adoption, implementation and maintenance. Reach captured engagement in screening, referral and legal services; effectiveness assessed preliminary outcomes such as HIV care engagement, viral suppression and quality of life; adoption examined integration into routine workflows; implementation assessed fidelity to core MLP components; and maintenance evaluated early indicators of sustainability. The CFIR framework complemented RE‐AIM by examining multilevel determinants of implementation, including organizational culture, leadership engagement, resource availability, intervention adaptability and external partnerships. CFIR‐informed measures assessed collaboration, communication, roles and responsibilities, and implementation milestones to identify key barriers and facilitators.

Data collection integrated quantitative and qualitative methods. Surveys assessed feasibility, acceptability, adoption and sustainability, while interviews and fieldnotes captured implementation experiences, contextual influences, adaptations and cross‐sector collaboration. Together, RE‐AIM and CFIR provided a rigorous mixed‐methods approach to understanding both MLP outcomes and the factors influencing successful implementation and sustainability across diverse settings.

### 2.7 Community Engagement

Each study included a Community Advisory Board (CAB) and Scientific Advisory Board (SAB) to ensure meaningful engagement of individuals with lived experience and interdisciplinary experts. CABs included PLWH, community advocates and service providers, while SABs comprised experts in HIV, public health, law and social work. Board members contributed to the development of screening tools, training curricula, evaluation measures and implementation strategies. Consistent with prior research highlighting the value of community engagement in improving health outcomes and intervention relevance, CABs provided critical insights into the lived experiences of PLWH navigating healthcare and legal systems, while SABs ensured interventions remained evidence‐based and responsive to community needs [49, 50, 51, 52, 53, 54, 55, 56, 57, 58].

### 2.8 Recruitment and Retention

A multi‐pronged recruitment and retention strategy was implemented across all sites. Recruitment combined community‐based and digital outreach through clinics, social service agencies, case managers, peer volunteers and targeted social media campaigns to reach diverse populations affected by HIV and HHLNs. Retention efforts included participant‐preferred communication (text, email or phone), privacy‐protected reminders, ongoing follow‐up by project coordinators and financial compensation for study participation. Consistent with CONSORT recommendations, these strategies promoted equitable access, minimized attrition and supported sustained engagement across study populations [59, 60].

### 2.9 Ethical Considerations

Across all three studies, individual randomization was not employed due to ethical concerns about assigning participants within the same clinic to receive legal aid services versus referrals. Instead, sites were randomized or assigned at the organizational level to ensure equitable access to legal support. All study participants provided informed consent prior to participation in the study.

Institutional Review Board (IRB) approvals were obtained from the University of Central Florida for MLP 1, WIRB‐Copernicus Group (WCG) IRB for MLP 2, and both Temple University and the University of Central Florida for MLP 3. For MLP 1 and MLP 3, additional oversight was provided by the University of Central Florida Data and Safety Monitoring Board (DSMB). For MLP 2, oversight was provided by a separate DSMB responsible for monitoring study conduct and participant protections.

## 3 Results

### 3.1 HHLNs Identified

Across the three MLP studies, 928 participants were enrolled between 2022 and 2026. RE‐AIM reach findings demonstrated the ability of MLPs to engage medically underserved populations disproportionately affected by HIV, structural inequities and legal vulnerabilities. High enrolment and screening completion rates supported the feasibility and acceptability of integrating HHLN screening and legal services into HIV care settings. In Orlando (MLP 1), 64 formerly incarcerated PLWH were enrolled and screened. The most common legal needs involved criminal record expungement, housing instability and disability benefits. Attorney case summaries indicated that approximately half of cases were successfully resolved, while the remainder required referral to external legal partners, particularly for immigration and criminal matters. Participants described legal barriers as major sources of stress that negatively affected healthcare engagement and financial stability. In MLP 2, 662 participants were enrolled across six primary care centres in San Juan, New York City and Philadelphia serving predominantly Latinx and migrant communities. Most participants reported at least one unmet legal need related to immigration, public benefits or housing. Common concerns included naturalization, asylum, U‐visa petitions, Medicaid and Social Security access, food assistance, housing repairs and eviction prevention. Qualitative findings suggested that legal uncertainty, discrimination and fear of deportation contributed to barriers to care engagement and psychological well‐being. In Philadelphia (MLP 3), 202 participants were enrolled and screened. Common legal concerns included advance care planning, unsafe housing conditions, eviction threats, workplace discrimination and other civil legal matters. Participants also reported stigma and discrimination in healthcare and employment settings. Across all sites, recurring HHLNs included housing instability, public benefit denials, immigration‐related concerns, advance care planning needs, criminal record expungement and discrimination.

### 3.2 Impact of MLPs on HIV Care and Well‐Being

#### 3.2.1 Participant Experiences and Perceived Benefits

Participants consistently described the MLP as both a practical and emotional source of support, highlighting improvements in access to resources, empowerment and mental well‐being. Participants reported tangible benefits from their engagement with the MLPs, including improved access to public benefits, reduced housing insecurity and greater confidence in navigating complex systems. Many participants described feeling empowered and supported through the process of addressing their legal challenges. One participant reflected, “*He was doing a living will for me; he talked to my kids. He was one of the greatest persons, every step was done*.” Another noted, “*They explained all aspects of setting up my living will, it gave me a settled peace of mind*.” Several participants described a newfound sense of control and calm after receiving legal assistance: “*Everything is calm, and little by little they are helping me…*.” Others emphasized how addressing legal barriers directly improved their mental health and self‐efficacy. As one client shared, “*Good experience, it helped me in all ways. I learned things about my legal needs, and it helped me with my mental health*.” Even participants who encountered systemic barriers, such as the inability to expunge a criminal record under state law, expressed appreciation for having received accurate information and compassionate guidance: “*I needed to get a misdemeanor off my record… it discouraged me a little, but at least I know what to expect*.”

#### 3.2.2 Psychological and Emotional Impact of MLP Participation

Beyond clinical outcomes, participants repeatedly described the psychological and emotional benefits associated with resolving HHLNs through the MLP. Participants consistently explained that addressing legal stressors reduced anxiety, fostered emotional stability, and improved their ability to focus on their health and HIV treatment. Many described legal concerns related to housing instability, benefits disputes, advance care planning and unresolved civil matters as persistent sources of stress that consumed emotional and cognitive energy. As one client summarized, “*At least I don't have to think about my health directives anymore*.” Participants emphasized that having legal and financial concerns actively addressed alleviated chronic worry and allowed them to redirect attention towards medication adherence, self‐care and overall well‐being.

Several participants highlighted the reassurance and peace of mind that came from working with trusted attorneys who explained complex legal processes in clear, accessible language. One participant shared, “*I felt comfortable; he answered my questions. He helped, I didn't feel nervous or anything. I appreciated it*.” Participants frequently noted that understanding their legal rights and available options reduced uncertainty and enhanced their sense of control. As one individual reflected, “*Good communication helped me make better choices*,” underscoring the relationship between legal literacy, empowerment and psychological relief.

Participants also emphasized the importance of compassionate and trusting attorney‐client relationships in shaping their overall experience and mental well‐being. One participant stated, “*He was great….He helped me get my housing back*.” Others similarly described their attorneys as approachable, attentive and trustworthy: “*[Attorney] is a great lawyer. He really listens… He answered all the questions. He's easy to talk to and has helped many people*.” These supportive relationships fostered trust, validation and a sense of partnership that extended beyond legal problem‐solving alone.

For many participants, simply being heard, informed and consistently supported was itself therapeutic. One participant noted, “*It was an incredible experience. He explained what we can and can't do and gave me information to follow up. It was excellent*.” Another added, “*Everything is fine, no issues. It just makes things easier to understand and eases concerns*.” These accounts illustrate how clear communication, responsiveness, and ongoing follow‐up reduced anxiety and contributed to emotional stability.

Importantly, participants described emotional benefits even when legal issues remained unresolved or ongoing. One individual shared, “*I met with him and he is still working on my legal name change. He is good*.” Another commented, “*He told me, ‘Don't worry, you'll be alright.’ He was able to help me*.” These experiences suggest that the presence of consistent advocacy, reassurance and trusted support may itself contribute to improved self‐confidence, optimism and psychological well‐being.

#### 3.2.3 Provider Perspectives on MLP Integration

Providers similarly emphasized that embedding legal services within healthcare settings strengthened patient engagement and continuity of care. Providers across sites reported positive perceptions of the MLP's impact, particularly its role in improving patient engagement, retention in care and access to supportive resources. One provider commented, “*Providing a new service will hopefully increase participants’ linkage to care and other patient assistance programs, support groups, and reduce recidivism as formerly incarcerated individuals will have a medical home and people who care about them*.” Another noted, “*The barriers facing the formerly incarcerated and those living with HIV are numerous, and the path to relief is fraught with inequities. Having a trusted legal partner brings me peace of mind, and my clients know the guidance is coming from a reliable source*.” Providers also observed improvements in patient empowerment: “*Patients are making informed decisions by knowing their legal rights, resulting in better outcomes in all aspects of their lives, including health*.”

#### 3.2.4 HIV Care Continuum Outcomes

In addition to qualitative findings, quantitative analyses from completed trials demonstrated promising effects on HIV‐related clinical outcomes. While MLP 1 and 2 are ongoing, with analyses of long‐term HIV care and quality‐of‐life outcomes forthcoming, the Philadelphia (MLP 3) trial has been completed. Kaplan–Meier survival analyses and logistic regression models assessed time to viral suppression and the likelihood of achieving an undetectable viral load at 3 and 6 months. At the 3‐month follow‐up, MLP participants were significantly more likely to achieve viral suppression compared to those receiving standard care (OR = 2.42, 95% CI: 1.06–5.49, *p* = 0.03). Although the 6‐month odds ratio declined (OR = 2.28, 95% CI: 0.79–6.57), the effect remained clinically meaningful but not statistically significant. Detailed findings have been reported elsewhere [61].

Collectively, these findings suggest that MLPs may function not only as structural interventions addressing HHLNs, but also as relational and psychosocial interventions that strengthen patients’ ability to engage in HIV care. Overall, participants described their engagement with MLPs as an experience that “*helped me in all ways… It helped me with my mental health*,” emphasizing how resolving legal stressors and gaining access to trusted advocates eased psychological distress. Across sites, the evidence suggests that reducing legal insecurity not only alleviates anxiety but also strengthens patients’ capacity to engage in HIV care and sustain positive health behaviours.

### 3.3 Contextually Responsive MLP Implementation Strategies

Although the three MLP initiatives shared core implementation components, strategies varied across sites based on local context, organizational readiness, legal infrastructure, policy environments and population needs. In Orlando, implementation emphasized legal issues affecting formerly incarcerated PLWH, including criminal record expungement, Social Security disability benefits, advance directives, housing stabilization and landlord–tenant disputes. These strategies addressed structural barriers to community re‐entry, including unstable housing, employment challenges and disruptions in healthcare access. In New York City, implementation was shaped by collaboration with a non–Legal Services Corporation (LSC)‐funded legal aid organization, enabling immigration‐related legal services often unavailable in LSC‐funded settings, including asylum, U‐visas, adjustment of status and naturalization. In San Juan, implementation increasingly focused on ageing populations through advance directives, powers of attorney and benefits navigation, reflecting Puerto Rico's demographic shifts, Hurricane Maria's long‐term effects, economic instability and healthcare workforce shortages. Strategies also emphasized culturally responsive bilingual services and community‐centred engagement. In Philadelphia, longstanding integration of legal services within HIV organizations facilitated preventive legal education, including “Know Your Rights” workshops, legal name changes for transgender individuals, advance care planning and legal literacy programming. Together, these contextually responsive strategies enhanced feasibility, trust, reach, acceptability, and sustainability across diverse healthcare and community settings.

### 3.4 Interdisciplinary Collaboration

MLPs fostered collaboration among healthcare, social service and legal partners, reducing provider burden while strengthening patient trust, engagement and continuity of care. Co‐located models enabled warm handoffs and coordinated responses to HHLNs, creating sustainable cross‐sector networks that addressed upstream determinants of health. For example, in Philadelphia, the partnership among The Philadelphia AIDS Consortium, Newlands Health and the Legal Clinic for the Disabled expanded HIV care through preventive legal workshops on housing, family law, advance care planning and legal name changes, illustrating the evolution of MLPs from reactive to preventive interventions. In Puerto Rico, the partnership between HealthProMed and Servicios Legales de Puerto Rico established the island's first known MLP, expanding access to integrated legal and healthcare services for medically underserved communities.

A summary of findings across the three MLP studies is presented in Table 2.

**TABLE 2:** Summary of findings across three medical‐legal partnership studies.

Domain	MLP 1: Orlando, FL	MLP 2: San Juan, PR/NYC/Philadelphia	MLP 3: Philadelphia, PA	Cross‐site findings
**Reach (RE‐AIM)**	64 formerly incarcerated PLWH enrolled and screened	662 Latinx and migrant participants enrolled across six primary care centres	202 PLWH enrolled and screened	A total of 928 participants have been enrolled to date across the three MLP studies, demonstrating successful engagement of medically underserved populations experiencing substantial legal, social and structural vulnerabilities.
**Clinical outcomes**	Ongoing	Ongoing	3‐month viral suppression significantly higher (OR = 2.42, 95% CI: 1.06–5.49, *p* = 0.03); effect persisted but was non‐significant at 6 months	MLP participation associated with faster viral suppression and improved care retention
**Mental health and empowerment outcomes**	“He talked to my kids… every step was done.” “Everything is calm and little by little they are helping me.”	“Good experience, it helped me in all ways. I learned things about my law needs and it helped me in mental health.”	“It gave me a settled peace of mind.” “At least I don't have to think about my health directives anymore.”	Participants described reduced anxiety, improved confidence, empowerment, and greater trust in healthcare and legal systems.
**Legal service outcomes**	Approximately 50% of cases closed successfully; remaining cases referred to external legal partners	Majority received immigration or benefits‐related support; some cases ongoing	High uptake of legal services for advance care planning and housing matters; expansion of preventive legal education workshops	High engagement in legal services across sites, with successful brief‐service interventions improving housing and benefits access.
**Adoption (RE‐AIM)**	Legal screening integrated into HIV care workflows	Strong organizational uptake across six primary care centres	High provider participation and integration of legal services	Broad adoption across healthcare and legal organizations.
**Implementation (RE‐AIM)**	High fidelity; strong leadership support; some resource constraints	High training completion; strong organizational readiness and adaptation across sites	Strong fidelity to intervention delivery	Core MLP components were implemented consistently across settings.
**Maintenance (RE‐AIM)**	Sustainability planning underway	Community‐engaged sustainability efforts in development	Sustainability challenges as funding cycles ended	Long‐term financing and institutionalization remain key priorities.
**CFIR facilitators**	Leadership engagement; interdisciplinary collaboration	Organizational readiness; community trust; bilingual service delivery	Established partnerships and co‐located services	Leadership buy‐in, interdisciplinary collaboration, organizational readiness and client‐provider trust facilitated implementation.
**CFIR barriers**	Staff turnover, resource constraints, immigration fears and recruitment challenges	Immigration fears and recruitment challenges	Workforce capacity and sustainability concerns	Workforce turnover, policy instability, funding uncertainty and recruitment of marginalized populations posed challenges across sites.
**Most common HHLNs**	Criminal record expungement; housing instability; benefit denials	Immigration concerns (naturalization, asylum, U‐visas); benefits access; housing repairs and eviction prevention	Advance care planning; unsafe housing; workplace discrimination	Shared HHLNs included housing instability, benefit denials, immigration barriers, criminal record expungement and discrimination.
**Provider perspectives**	“Participants will have a medical home and people who care about them.”	“A trusted legal partner brings me peace of mind and ensures reliable guidance.”	“Patients are making informed decisions by knowing their legal rights.”	Providers reported improved engagement, retention, care coordination and interdisciplinary teamwork.
**Interdisciplinary collaborations**	Collaboration among community‐based organizations, health centres and legal aid partners	Partnerships among primary care centres and legal service providers across three cities	TPAC, Newlands Health and Legal Clinic for the Disabled; preventive legal education clinics	Multi‐sector partnerships strengthened networks addressing structural determinants of HIV outcomes.
**Key lessons learned**	Addressing legal barriers supported stability and engagement in care	Immigration‐related legal insecurity emerged as a major structural challenge	Preventive legal education promoted empowerment and proactive planning	Structural, cross‐sector interventions show promise for addressing HHLNs and advancing health equity among PLWH.

Abbreviations: CFIR,  Consolidated Framework for Implementation Research; CI, confidence interval; FL, Florida; HHLNs, health‐harming legal needs; HIV, human immunodeficiency virus; MLP, medical‐legal partnership; NYC, New York City; OR, odds ratio; PA, Pennsylvania; PLWH, people living with HIV; PR, Puerto Rico; RE‐AIM, Reach, Effectiveness, Adoption, Implementation, and Maintenance; TPAC, The Philadelphia AIDS Consortium.

### 3.5 Equity‐Centred Implementation Framework for Medical‐Legal Partnerships in HIV Care

Cross‐study implementation findings and lessons learned informed the development of the equity‐centred implementation framework for medical‐legal partnerships in HIV care (Figure 1). Emerging iteratively through synthesis of implementation experiences across sites and populations, the framework integrates principles of implementation science, equity and community engagement to guide evaluation of MLPs across clinical, legal and structural domains. Guided by RE‐AIM, CFIR and Proctor's implementation outcomes, the framework outlines key inputs, activities, outputs and outcomes, including metrics for reach, fidelity, effectiveness, cost and sustainment, while emphasizing contextual determinants and equity‐focused analyses. The framework also incorporates pragmatic mixed‐methods approaches, CAB/SAB governance and established reporting standards (CONSORT, StaRI, TIDieR and CHEERS) to support rigorous evaluation, replication, adaptation, scaling and sustainability of MLP interventions. Although developed within HIV‐focused MLPs, the framework may be adapted for use across other healthcare settings, disease areas and populations.

**FIGURE 1:**
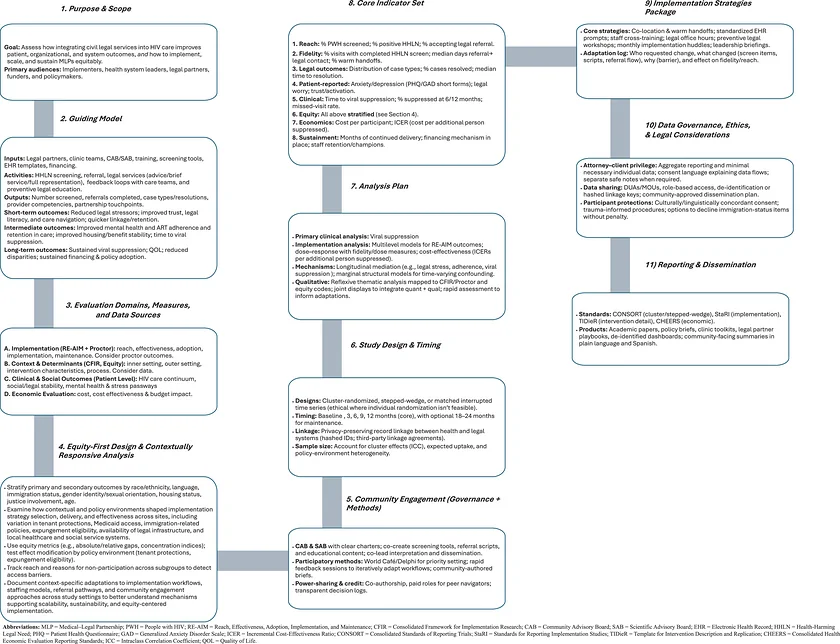
Equity‐centred implementation framework for medical‐legal partnerships in HIV care.

## 4 Discussion

The findings from this multi‐site programme of MLP studies underscore the potential of legal–medical integration as a structural intervention to improve HIV outcomes and reduce inequities across diverse populations. By embedding legal services directly within HIV care and primary care settings, MLPs were designed to address select upstream social and legal determinants that may impede engagement along the HIV care continuum. Across Orlando, Philadelphia, San Juan and New York City, the interventions proved feasible, acceptable and effective at identifying and addressing HHLNs, including housing instability, benefit denials, immigration barriers and criminal record expungement, that are well‐documented drivers of poor HIV outcomes [7, 8, 62, 63].

This article provides cross‐study synthesis of implementation experiences, preliminary outcomes and lessons learned across three federally funded MLP initiatives spanning four jurisdictions. Rather than describing study designs or planned methodologies, this manuscript integrates quantitative implementation indicators, participant and provider perspectives, attorney case summaries, and preliminary clinical findings to identify common implementation strategies, contextual adaptations, barriers, facilitators and cross‐site patterns. In addition, this manuscript introduces the equity‐centred implementation framework for MLPs in HIV care, which emerged from synthesis of findings across studies and multi‐site stakeholder consensus activities. The framework represents a novel contribution by integrating implementation science, health equity and community‐engaged principles into a pragmatic model intended to guide future implementation, evaluation, adaptation, and scale‐up of MLPs in HIV care and other healthcare settings.

These findings add to a growing body of evidence supporting the potential role of structural interventions in improving HIV prevention and care outcomes [61, 64, 65]. The completed Philadelphia trial (MLP 3) further demonstrates measurable clinical impact: participants receiving MLP services were significantly more likely to achieve viral suppression at 3 months compared to those receiving standard care. Although the 6‐month effect was not statistically significant, the consistent direction of the findings indicates that addressing HHLNs may accelerate time to viral suppression.

Participants’ narratives of reduced stress, improved mental well‐being and renewed focus on health following the resolution of legal problems align with the existing literature, which shows that alleviating social and legal issues can improve mental health outcomes [66, 67, 68]. The qualitative findings provide valuable insight into the psychosocial mechanisms underlying these improvements. Participants frequently described feeling “calmer,” “less stressed” and “more confident” after receiving legal support. Such emotional relief may translate into improved adherence behaviours and engagement in care. The reported therapeutic value of being “heard” and “taken seriously” by attorneys and providers points to the relational dimensions of legal advocacy, an often‐overlooked yet critical element of structural care.

The application of RE‐AIM and CFIR frameworks provided an empirically grounded understanding of the conditions necessary for successful MLP implementation. Leadership engagement, organizational readiness and interdisciplinary collaboration emerged as key facilitators, consistent with the implementation science literature, emphasizing the importance of inner‐setting factors and the implementation climate for adoption and sustainability [69, 70, 71]. The MLPs demonstrated that co‐location and cross‐sector collaboration, among medical providers, social workers and legal advocates, reduce provider burden, streamline service delivery and strengthen patient trust.

The long‐standing partnership between the Philadelphia AIDS Consortium, New Lands Health and the Legal Clinic for the Disabled illustrates how sustained collaboration can evolve from addressing acute crises to delivering preventive legal education, fostering community empowerment and reducing future legal burden. Similarly, the formation of Puerto Rico's first MLP and the Tri‐City Symposium on Health‐Harming Legal Needs highlight the value of knowledge exchange across jurisdictions. This type of multi‐sectoral collaboration exemplifies the *whole‐systems* approach that is increasingly advocated within HIV implementation science and social medicine [72, 73].

Despite their demonstrated effectiveness, MLPs face significant sustainability challenges. Long‐term integration into healthcare financing, through Medicaid reimbursement, managed care contracts or value‐based care incentives, will be essential for scalability. Embedding legal screening within electronic health records and quality improvement systems can further institutionalize the model and enhance accountability.

From an equity perspective, the MLPs also highlight the need for culturally responsive and linguistically competent implementation. Spanish‐speaking participants and other immigrant populations often experience compounding barriers, including language isolation, documentation concerns and fear of deportation, that limit access to care. Consistent with existing research, these findings affirm that cultural humility and trust‐building are prerequisites for engaging Latino and migrant populations [74, 75, 76].

Workforce development represents another key pillar of sustainability. Training clinicians, legal professionals and peer navigators to identify HHLNs and hiring from within communities disproportionately impacted by HIV can enhance both reach and cultural resonance. This aligns with national calls to strengthen the implementation science workforce through interdisciplinary, community‐engaged training models [77, 78].

The results of these studies have laid the foundation for the next generation of MLP research, including large‐scale hybrid effectiveness–implementation trials such as the proposed Borikén MLP in Puerto Rico and the Mobile Medical‐Legal Partnership initiative in Central Florida. These models represent an evolution of MLPs from fixed‐site clinics to flexible, community‐based delivery systems that integrate health, legal and social services. Future research should examine mediating mechanisms, such as stress reduction, social capital and legal empowerment, that link MLP participation to biological outcomes, such as viral suppression and immune recovery. Longitudinal analyses will also be essential to determine the durability of these effects and to guide economic evaluations of cost‐effectiveness.

This study has several limitations. First, causal inference is limited by the heterogeneity of study designs, including a cluster‐randomized trial, a before–after pilot and an ongoing hybrid implementation trial. Site‐level assignment reduced ethical concerns but may have introduced selection bias and unmeasured confounding. Second, some outcomes relied on self‐report and may be subject to recall or social desirability bias. Third, generalizability may be limited because the studies were conducted primarily in urban settings with established legal partners, and implementation occurred within rapidly changing policy and funding environments that affected recruitment, staffing and participant experiences. Fourth, variation across sites in staffing, co‐location, legal service delivery and caseloads may have influenced outcomes despite high implementation fidelity. Fifth, limited follow‐up periods and sample sizes constrain clinical inference, particularly because many legal matters require extended time to resolve. Sixth, while RE‐AIM and CFIR provided a strong implementation framework, they do not fully capture structural racism, power or justice; complementary equity‐focused frameworks may strengthen future evaluations. Finally, the equity‐centred implementation framework for medical‐legal partnerships in HIV care was informed by cross‐study findings but has not yet been prospectively tested. Future research should evaluate its application across diverse settings while examining long‐term outcomes, sustainability, economic impact and integration into routine healthcare financing systems.

## 5 Conclusions

Collectively, this programme of research demonstrates that MLPs are not only feasible and acceptable but also offer promise in addressing the structural and psychosocial barriers that perpetuate HIV disparities. By bridging healthcare and justice systems, MLPs embody a model of “justice as care,” positioning legal advocacy as an integral component of health equity. These findings advance the literature on structural interventions by showing that embedding legal services within care settings may improve engagement, reduce stress and accelerate viral suppression among PLWH. As health systems and policymakers seek innovative strategies to meet the goals of the Ending the HIV Epidemic initiative, the evidence presented here suggests that MLPs offer a powerful, sustainable and equity‐driven approach to achieving those aims.

## Author Contributions

Omar Martinez conceptualized the study, led the overall design and analysis, and drafted the initial version of the manuscript. Guillermo Prado, Kathryn M. Nowotny, Robin Davison, Cui Yang, Samantha Morton, Ashley French, Edda Santiago Rodriguez, Souhail Malave‐Rivera, Carlos E. Rodriguez‐Diaz, Florence Momplaisir, Elena Cyrus, Mallory O. Johnson and Miguel Muñoz‐Laboy contributed to the study design, interpretation of findings and critical revisions for important intellectual content. The integration of implementation science frameworks and the conceptualization of the evaluation approach were led by Omar Martinez, Guillermo Prado, Edda Santiago Rodriguez, Souhail Malave‐Rivera, Carlos E. Rodriguez‐Diaz and Miguel Muñoz‐Laboy.

## Conflicts of Interest

The authors declare no conflicts of interest.

## Supporting information




**Supplement A**: Legal Problem Macro Categories.

**Supplement B**: Attorney Case Summary.

**Supplement C**: Tri‐City Symposium Agenda.

## Data Availability

The data that support the findings of this study are available on request from the corresponding author. The data are not publicly available due to privacy or ethical restrictions.
