# Supplementary material for: Medical‐Legal Partnerships as Structural Interventions: Integrating Implementation Science and Community‐Engaged Research to Advance HIV Care
Source: J Int AIDS Soc. 2026 Oct 8;29(Suppl 5):e70191. doi: 10.1002/jia2.70191 (PMC13650414; doi:10.1002/jia2.70191)
Supplement: Supplementary file 1 [file JIA2-29-e70191-s001.docx]

**OPAHL-MC Legal Services Summary Form**

**REVISED – January 2026**

|  |  | **Data to be entered by [LEGAL ORG]** | **Instructions** | **Definitions** |
| --- | --- | --- | --- | --- |
| **Case status** | A. Open or closed? | Open __  Closed __  Declined/rejected at intake stage __ |  | A client’s case remains **open** if even one legal matter is still in-progress.  A client’s case is **closed** only if *all* legal services have ceased and the legal services provider has documented the engagement’s closing for the client in writing.  A client’s case has been **declined/rejected** if it was determined to be out-of-scope. See Section F below, which asks you to indicate what type of “out-of-scope” the case was (v.1 vs. v.2). |
| **Client informa-tion** | B. Study ID number |  | When the attorney is preparing to complete a batch of these forms, contact the study team. The study team will send the attorney a linked (a) name and (b) Study ID number. When the attorney fills out this form, only include the Study ID number, not the client’s name, and send the completed form back to the study team. This promotes de-identification of study enrollees during the data analysis phase. | N/A |
|  | C. # of individuals living in household |  | N/A | N/A |
| **Intake interview (or Initial Interview)** | D. Date(s) of interview conducted by attorney |  | N/A | N/A |
|  | E. Legal concern(s) identified based on interview conducted by attorney | (1) | List one concern per row. using standard [LEGAL ORG] case management descriptor **– including any applicable Legal Problem Code or Special Problem Code.** | N/A |
|  |  | (2) |  |  |
|  |  | (3) |  |  |
|  |  | (4) |  |  |
|  |  | (5) |  |  |
| **Legal services summary** | F. Legal concern(s) identified at intake stage that are  (1) outside the scope of [LEGAL ORG’s] funded work or  (2) outside the scope of [LEGAL ORG’s] priorities or capacity. | (1) | List one concern per row, using standard [LEGAL ORG] case management descriptor **– including any applicable Legal Problem Code or Special Problem Code.**  **Then add a notation indicating whether the out-of-scope legal concern was**   - **Out-of Scope v.1 (prohibited by funding terms)  *or*** - **Out-of-Scope v.2 (beyond the organization’s priority areas or current capacity).**   If 3+ legal concerns identified at intake stage were outside [LEGAL ORG’s] capacity and/or expertise, add new rows below (e.g., H(4), H(5), etc.). | N/A |
|  |  | (2) |  |  |
|  |  | (3) |  |  |
|  | G. Re: F, describe any efforts you made to facilitate client connection to alternate legal providers for those concern(s), such as referrals. |  | Share this description in free text. | “You”: The person completing this form as well as anyone else employed by [LEGAL ORG]. |
|  | H. Specific legal services *offered* by [LEGAL ORG] to client | (1) | List one form of *offered* legal service per row, using standard [LEGAL ORG] case management descriptor of service **– including any applicable service code or terminology (e.g., “Brief service”, “Extensive Service”, etc.).**  If there were 3+ forms of legal service, add new rows below (e.g., H(6), H(7), etc.). | “Legal services”: The full range of activities undertaken by [LEGAL ORG] when providing civil case assessment and legal representation services to individual clients. These include, but are not limited to, advising a client on their legal rights and responsibilities, delivering brief advice and service, and full representation in court or administrative proceedings. |
|  |  | (2) |  |  |
|  |  | (3) |  |  |
|  |  | (4) |  |  |
|  |  | (5) |  |  |
|  | I. Specific legal services *directly provided* by [LEGAL ORG] to client | (1) | List one form of *directly-provided* legal services per row, using standard [LEGAL ORG] case management descriptor of service **– including any applicable service code or terminology (e.g., “Brief service”, “Extensive Service”, etc.).**  If 3+ forms of legal service were provided, add new rows below (e.g., I(4), I(5), etc.). | See above. |
|  |  | (2) |  |  |
|  |  | (3) |  |  |
|  | J. Describe your impressions of how [LEGAL ORG’s] direct provision of legal services to client impacted them |  | Share this description in free text.   Impressions can reflect anything on the continuum of positive v. negative v. neutral v. other. | N/A |
| **If case is closed:** | K. Date of closing letter (communication) that was transmitted to client |  | N/A | N/A |
|  | L. Estimated # of hours dedicated by [LEGAL ORG] to client’s case |  | Applies to all staff who contributed to the work, not simply the attorney dedicated to the study intervention. | N/A |

**[LEGAL ORG] representative who completed this form:	Date this form**
Name & Title: ______________________________________	**was completed:** _____________________

**Abbreviations:** ID = identification; MC = Monitoring and Coordination; N/A = not applicable; OPAHL = Organizational Partnerships for Healthy Living.
