# Supplementary material for: Medical‐Legal Partnerships as Structural Interventions: Integrating Implementation Science and Community‐Engaged Research to Advance HIV Care
Source: J Int AIDS Soc. 2026 Oct 8;29(Suppl 5):e70191. doi: 10.1002/jia2.70191 (PMC13650414; doi:10.1002/jia2.70191)
Supplement: Supplementary file 2 [file JIA2-29-e70191-s002.docx]

***Addressing durable health disparities through critical time legal interventions in medically underserved Latinx and migrant communitie*s**

**Agenda for Multisite Coordination Meeting**

**Condado, San Juan, Puerto Rico**

*February 5^th^ to 7th, 2026*

**Day 1: Thursday, February 5, 2026**

**Arrival to San Juan, Puerto Rico**

**Caribe Hilton**

**1 San Geronimo Street,**

**San Juan, PR, 00901**

***Meeting Room: Caribar Terrace***

**6pm	Welcome** (Carlos Rodriguez-Diaz, PhD)

- Opening remarks
- Meeting objectives
- Brief introductions around the room (name, affiliation, preferred dessert, one expectation from today’s meeting)

**7pm	Dinner and getting to know each other**

***_______________________________________________________________________________***

**Day 2: Friday, February 6, 2026**

***Room: Salon Del Mar***

**8:00am	Arrival to meeting room and light breakfast**

**8:30am	Objectives for the day** (Miguel Muñoz-Laboy, DrPH)

**8:45am	Legal Epidemiology: Landscape and Future Directions** (Omar Martinez, JD, MPH)

**9:15am	Cross-Site Highlights and Progress Updates** (Souhail Malave Rivera, PhD)

- Patient/client enrollment in MLP services and in legal treatment as usual (off-site referrals/referral sheet)
- Staff survey/assessment enrollment
- Timeline and milestones

***_______________________________________________________________________________***

**Differences and similarities** (Facilitator: Souhail Malave Rivera, PhD)

**9:30am	Panel: Integrating Legal Services at Partner Clinics**

- Philadelphia: Legal Clinic for the Disabled providing services at TPAC (Andrew Maude, JD)
- New York: LegalHealth (NYLAG) providing services at Betances Health Center (Gerry Ruiz, JD)
- San Juan: Servicios Legales de Puerto Rico providing services at Health ProMed (Lcdo. Ricardo Agrait Defilló, JD)

**10am	Panel: Recruitment and Retention – Onsite MLP Services**

- Betances Henry street (Evee Colon, MSWc)
- TPAC (Sandra Rodriguez)
- HealthProMed - Santurce (Milton Rodriguez, MPHE, DrPHc)

**10:30am	Panel: TAU - Panel: Recruitment and Retention – Referral-Based (TAU) Services**

- New Lands (Deja Alvarez)
- HealthProMed - Caparra (Milton Rodriguez, MPHE, DrPHc)
- Betances Bushwick (Luis Sanchez)

**11am	Panel: Staff - Implementation assessment, recruitment and retention issues**

- HealthProMed Santurce & Caparra (Milton Rodriguez, MPHE, PhDc)
- TPAC/New Lands (Sandra Rodriguez)
- Betances Henry St./Bushwick (Jessica Zambri, MSWc)

***_______________________________________________________________________________***

**Preliminary findings** (Facilitator: Carlos Rodriguez-Diaz, PhD)

**11:30am	Demographic typology of patients/clients at baseline** (Yareliz Diaz, PhD)

**11:50am	Health harming legal needs at baseline** (Ashley French, MA, PhDc and Amanda

Cruz Gerena, MS, M.Ed)

**12:10pm	Implementation process evaluation** (Jessica Zambri, MSWc)

***_____________________________________________________________________________***

**12:30pm	Lunch**

***_______________________________________________________________________________***

**From your Point of View: Forecasting Critical Issues** (Facilitator: Omar Martinez, JD, MPH)

**1:30pm	Panel: From your (Health) Point of View**

- Key or new challenges in providing health and social services in the territory of Puerto Rico (Heidy Lopez on behalf of Lcda. Ivonne Rivera, Executive Director of HealthProMed)
- Key or new challenges in providing health and social services in the state of New York (Evette M. Maduro, Chief Executive Officer, Betances Health Centers)
- Key or new challenges in providing health and social services in the state of Pennsylvania **(Deputy Executive Director, Deja Alvarez)**

**2:15pm	Panel: From your (Legal) Point of View**

- Key or new challenges in providing legal aid in the territory of Puerto Rico (Licenciada Hadassa Santini Colberg)
- Key or new challenges in providing legal aid in the state of New York (Julie Brandfield, JD)
- Key or new challenges in providing legal aid in the state of Pennsylvania (Theresa Brabson, JD)

***_____________________________________________________________________________***

**Brainstorming integration and sustainability of medical legal partnerships panel** (Facilitator: M. Muñoz-Laboy, DrPH)

**3pm	Setting up the discussion:** Case study: Preliminary analysis of staff surveys in New York site **(Yareliz Diaz, PhD and Jessica Zambri, MSW)**

**3:20pm	*Questions for the tables*** (Facilitators: Heidi Lopez, Rose Velez, Amanda Cruz-Gerena,

Evee Colon, Alejandro Figueroa, Gelismin Marrero, Sandra Rodriguez)

- What are the challenges of integrating health, social and legal services?
- What are the most effective ways of balancing different types of services?
- Which model or models are most feasible from your point of view?
- What are sustainable models for building medical legal partnerships?

**4pm	Reports back from the tables by facilitators and group discussion**

**4:30pm	Discussants from the Macro-Point of View** (Bethany Hamilton, JD, NCMLP, Enerrie Escoto, Betances, and, Dr. Migdalia Perez, HealthPromed)

***_____________________________________________________________________________***

**4:50pm	*Other outstanding issues in the implementation of MLP*** (Bethany Hamilton, JD,

NCMLP)

***_____________________________________________________________________________***

**5:15pm	Closing Reflections of the Day** (Maria Torres, PhD)

***_______________________________________________________________________________***

**Dinner on your own.**

**Day 3: Saturday, February 7, 2025**

***Room: Salon Del Mar***

**This will be an interactive morning. We are asking each team to meet with both Dr. Juan de la Cruz (to discuss micro-costing, a technique to evaluate the costs) and the EHR team (to discuss the electronic health records process and clarify legal quantitative data extraction). EHR team includes: Dr. Yareliz Diaz, Dr. Carlos Rodriguez Diaz, & Epiphany Adams, MSW, PhDc)**

**Agenda for Philadelphia team (Health, legal providers and researchers)**

8:30-9am	Breakfast and Coffee

9-9:30am	Meeting with Dr. DelaCruz

9:30-10am	Meeting with EHR team

10-10:30am	Quiet time, break to check out

**Agenda for New York team (Health, legal providers and researchers)**

8:30-9am	Breakfast and Coffee

9-9:30am	Quiet time, break to check out

9:30-10am	Meeting with Dr. DelaCruz

10-10:30am	Meeting with EHR team

**Agenda for Puerto Rico team (Health, legal providers and researchers)**

8:30-9am	Breakfast and Coffee

9-9:30am	Meeting with EHR team

9:30-10am	Quiet time, break to check out

10-10:30am	Meeting with Dr. DelaCruz

***_______________________________________________________________________________***

**10:30am	Site-specific goals for 2026** (Facilitator: Dr. Carlos Rodriguez-Diaz)

**11am	Closing Remarks** (Omar Martinez, JD, Souhail Malave Rivera, Carlos

Rodriguez Diaz, Miguel Muñoz-Laboy)

- Acknowledgment of Participants
- Expression of Gratitude for Engagement and Contributions

**11:30am	Meeting ends**

**Abbreviations:** EHR = electronic health record; JD = Juris Doctor; MA = Master of Arts; M.Ed. = Master of Education; MLP = Medical-Legal Partnership; MPHE = Master of Public Health Education; MS = Master of Science; MSW = Master of Social Work; MSWc = Master of Social Work candidate; NCMLP = National Center for Medical-Legal Partnership; PhD = Doctor of Philosophy; PhDc = Doctor of Philosophy candidate; PR = Puerto Rico; TAU = treatment as usual; TPAC = The Philadelphia AIDS Consortium.
